# Revision of the Choices Nutrient Profiling System

**DOI:** 10.3390/nu18020258

**Published:** 2026-01-14

**Authors:** Herbert Smorenburg, Katrina R. Kissock, Eleanor J. Beck, Pulkit Mathur, Bruce Hamaker, Lauren Lissner, Mario R. Marostica, Ngozi Nnam, Hidemi Takimoto, Annet J. C. Roodenburg

**Affiliations:** 1 Choices International Foundation, 6981 CH Doesburg, The Netherlands; 2 The George Institute for Global Health, University of New South Wales, Randwick, NSW 2031, Australia; kkissock@georgeinstitute.org.au; 3 School of Health Sciences, Faculty of Medicine & Health, University of New South Wales, Sydney, NSW 2052, Australia; e.beck@unsw.edu.au; 4 Department of Food & Nutrition and Food Technology, Lady Irwin College, University of Delhi, New Delhi 110001, India; pulkit.mathur@lic.du.ac.in; 5 Department of Food Science, Purdue University, West Lafayette, IN 47907, USA; hamakerb@purdue.edu; 6 Institute of Medicine, School of Public Health and Community Medicine, Sahlgrenska Academy, University of Gothenburg, P.O. Box 469, 405 30 Gothenburg, Sweden; lauren.lissner@gu.se; 7 Department of Food Science and Nutrition, School of Engineering, Universidade Estadual de Campinas, Campinas, São Paulo 13083-862, Brazil; mmarosti@unicamp.br; 8 Department of Nutrition & Dietetics, University of Nigeria, Nsukka 410001, Nigeria; ngnnam@yahoo.com; 9 National Institute of Health and Nutrition, National Institutes of Biomedical Innovation, Health and Nutrition, Osaka 566-0002, Japan; thidemi@nibn.go.jp; 10 Department of Nutrition and Health, HAS University of Applied Sciences, 5200 MA’s-Hertogenbosch, The Netherlands; a.roodenburg@has.nl

**Keywords:** front-of-pack nutrition labeling, nutrient profiling, choices 5-level criteria, plant-based alternatives, meat alternatives, dairy alternatives, milk alternatives equivalence criteria, non-sugar sweeteners, non-nutritive sweeteners, food policy, nutrition policy

## Abstract

Background/Objectives: Poor dietary habits are a major contributor to non-communicable diseases (NCDs), the leading cause of mortality worldwide. To promote healthier eating, governments and stakeholders have implemented various nutrition policies, including front-of-pack nutrition labeling (FOPNL). The Choices International Foundation (Choices), through its criteria, supports these efforts through its standardized nutrient profiling system (NPS). Originally developed to underpin a positive FOPNL logo, in 2021, the criteria were expanded into a globally oriented five-level profiling system covering 23 basic and 10 discretionary food groups, addressing key nutrients such as trans-fatty acids, saturated fat, sodium, sugar, fiber, and energy. To ensure continued scientific relevance, the Choices criteria are periodically reviewed by an independent International Scientific Committee (ISC). Methods: This paper presents the 2025 revision of the Choices criteria, focusing on priority areas identified through stakeholder consultation and recent scientific developments. Results: Key updates include the introduction of nutrient-based equivalence criteria for plant-based alternatives to meat and dairy, based on protein and selected micronutrient thresholds. Non-sugar sweeteners (NSSs) were newly included as a factor that lowers a product’s health classification and makes it ineligible for a positive FOPNL. Additionally, the industrially produced trans-fatty acid (iTFA) criteria were revised and aligned with the latest World Health Organization (WHO) recommendations, improving both technical feasibility and policy coherence. While options for incorporating whole-grain and micronutrient criteria were explored, these were not included in the current revision. Conclusions: The 2025 update system enhances the scientific rigor, policy alignment, and global applicability of the Choices system. By providing a harmonized and evidence-based tool, it aims to support national policies that foster healthier food environments and, ultimately, improve public health outcomes worldwide.

## 1. Introduction

Poor dietary habits are a major preventable risk factor for non-communicable diseases (NCDs) [1]. In 2021 alone, NCDs were responsible for at least 43 million deaths, accounting for 75% of all non-pandemic-related global mortality. Notably, 18 million of these deaths occurred before the age of 70, with 82% of these premature deaths taking place in low- and middle-income countries (LMICs) [2]. In response to this growing burden of diet-related NCDs and the persistent gap in adherence to dietary guidelines, interest in evidence-based nutrition policy tools has intensified globally. Among these tools, front-of-pack nutrition labeling (FOPNL) aims to provide consumers with clear, accessible information at the point of purchase to facilitate healthier choices [3]. Additional policies, including regulations to protect children from the harmful impact of food marketing [4], fiscal measures such as taxes and subsidies [5], and school food programs, are increasingly implemented to promote healthier diets.

A key component underpinning these strategies is the use of government-endorsed nutrient profiling systems (NPSs), which categorize foods based on their nutrition composition [6,7,8]. Most NPSs focus on nutrients to limit, with sodium, saturated fatty acids, and total sugars being included most frequently, and many also consider one or more nutrients to encourage (e.g., fiber and/or protein) [6]. However, differences in classification methodology, governance structures, and intended applications contribute to a diverse and often fragmented NPS landscape. For instance, while positive and graded FOPNL schemes are generally implemented on a voluntary basis, warning labeling is mandated through legislation [7]. Most NPSs have historically been designed to reduce the prevalence of diet-related chronic diseases in high-income countries, yet several LMICs have now developed or adapted their own models in response to local nutrition challenges [8]. For example, Malaysia, Thailand, and Indonesia have implemented the Healthier Choice Logo, a product-group-specific positive label with thresholds for nutrients to limit and encourage [9,10,11]. Similarly, Mexico, Uruguay, Peru, Colombia, and Argentina have implemented mandatory warning labels, adapted from the Chilean model, based on nutrient content and the presence of non-sugar sweeteners (NSSs) [12].

The Choices International Foundation (Choices) originally developed its NPS to identify foods eligible for a positive FOPNL, with the dual aim of guiding consumers and encouraging food reformulation [13]. In short, the system is based on product-group-specific nutrients, distinguishing between basic and discretionary (or non-basic) food categories. Products in basic food categories are expected to contribute positive nutrients to the diet and are included in food-based dietary guidelines (FBDGs), whereas discretionary foods are consumed for convenience or pleasure, but not for nutritional reasons. Thresholds for industrially produced trans-fat, saturated fat, sodium, added sugars, and fiber are derived from international nutrient recommendations and calibrated using food composition data. Compliance targets (20% for basic foods, 10% for discretionary foods) were used to ensure practical feasibility. Predictive validation studies demonstrated potential effects on dietary intakes [14,15,16]. The Choices criteria were designed to be adaptable across different national contexts to support their national positive logo programs. Various studies have shown the efficacy of Choices programs [17,18,19]. To ensure continued scientific relevance, the Choices criteria are periodically reviewed by an independent International Scientific Committee (ISC). Previous revisions in 2010, 2014, and 2018 have been documented by Van den Assum et al. [20].

In 2021, Choices extended its NPS to a five-level system to support not only positive but also graded and negative FOPNL, as well as other nutrition policies, such as marketing restrictions and fiscal policies [21]. This expanded system retained the foundational methodology but introduced additional tiers to classify food healthiness, from level 1 (healthiest) to level 5 (least healthy). The thresholds were derived using international food composition data and aligned with the World Health Organization (WHO) NPSs developed for restricting marketing to children. Validity assessments confirmed good alignment with dietary guidelines from diverse regions, including Europe, Africa, and Asia [21,22,23].

Other international NPSs such as the Health Star Rating (HSR), the Nutri-Score and the WHO Regional Office for Europe NPS have undergone similar updates [24,25,26]. These updates included, amongst others, adaptations for plant-based food and drinks, NSSs, and the tightening of existing thresholds.

The main objective of the Choices 2025 revision is to address concerns that were raised by stakeholders in a targeted consultation as part of the development process of the Choices 5-levels criteria [21]. Common reasons for questioning the Choices product groups included the classification of plant-based alternatives to milk as a discretionary food group and the absence of a food group for plant-based alternatives to meat. Some stakeholders expressed the need for also considering more positive nutrients beyond fiber. One stakeholder disagreed with the classification of both red and processed meat products as basic food products, despite being classified as probably carcinogenic by the International Agency for Research on Cancer [27]. Furthermore, some stakeholders expressed concerns about the validity of the database, which was used for interpolation purposes. Finally, questions were raised about the threshold levels, for either being too strict or too lenient.

The present paper describes the first revision of the Choices criteria since the introduction of the five-level system in 2021. It addresses feedback from stakeholders regarding key limitations of the 2021 version, including the lack of criteria for plant-based meat alternatives, the classification of non-dairy milk substitutes, and the absence of positive nutrients such as whole grains and micronutrients as qualifying components. Furthermore, the updates of the HSR and Nutri-Score programs and the renewed guidelines of the WHO, in particular their trans-fat guidelines and position on NSSs, were considered. The paper outlines the rationale, methodology, and outcomes of the 2025 revision, with particular focus on how it incorporates recent scientific evidence, aligns with international standards, and supports diverse nutrition policy applications. 

## 2. Rationale and Methodology

In the first half of 2022, shortly after the publication of the Choices five-level NPS, the ISC met twice to define the scope and process for the 2025 revision. Several topics were identified for further investigation, including classification and equivalence criteria for plant-based alternatives, the role of non-sugar sweeteners (NSSs), the inclusion of whole-grain and micronutrient criteria, the alignment of industrially produced trans-fat (iTFA) thresholds with WHO guidelines, the use of alternative food composition databases, and comparison with national FBDGs and other NPSs. The ISC decided to retain processed meat within the basic food groups—defined as foods contributing essential nutrients—as many FBDGs continue to recommend (processed) meat and meat substitutes as part of healthy and sustainable diets. The Choices criteria also differentiate between healthier and less healthy meat products, whereas some references associate carcinogenicity with all red and processed meats.

Several ISC-supervised research projects assessed the Choices NPS against national dietary guidelines and international profiling models. These studies showed good overall alignment with systems such as the Dutch Wheel of Five, the Nordic Keyhole, and Nutri-Score [22,23], with some differences reflecting local policy priorities rather than methodological issues. The research also highlighted areas for future development, including the potential relevance of micronutrient criteria—which require further analysis—and the feasibility of nutrient-based equivalence criteria for plant-based alternatives.

Based on these findings, recent international policy updates (e.g., HSR, Nutri-Score, WHO NPS), and internal deliberations, the ISC agreed to limit the scope of the 2025 revision to four core areas:

1. Classification and equivalence criteria for plant-based alternatives;
2. The position on NSSs;
3. Alignment of iTFA criteria with WHO guidelines; and
4. Consideration of whole-grain criteria.

### 2.1. Classification and Equivalence Criteria for Plant-Based Alternatives

The production of animal-source foods has well-documented environmental impacts, including high greenhouse gas emissions, the degradation of soil quality, the disruption of water systems, and the loss of biodiversity [28]. In light of growing environmental concerns, numerous organizations, including the EAT Lancet Commission, have advocated for a reduction in meat consumption and a shift towards an increased intake of plant-based foods [29]. This transition is further supported by broader cultural trends favoring sustainability, as well as growing evidence linking the consumption of processed meats to negative health outcomes [27]. As a result, consumer demand for plant-based alternatives to meat and dairy has been rising rapidly, with the U.S. market for plant-based foods alone valued at USD 8.1 billion [30].

Given these developments, there is a clear need to define how plant-based alternatives should be evaluated within NPSs. The Choices framework recognizes basic food groups as sources of essential nutrients and allows products, if compliant with the relevant nutrient thresholds in these product groups, to be positioned as “healthier” (Choices level 1 or 2) and eligible for health-related positioning. However, until now, plant-based milk alternatives have been classified as discretionary foods within the Choices system, as many such products do not naturally provide the expected levels of positive nutrients associated with dairy. As a result, they have been ineligible for health-related positioning.

Furthermore, the absence of dedicated product groups for plant-based meat or cheese alternatives has created ambiguity in how these products should be classified and assessed. Animal-based products typically provide important nutrients such as protein, calcium, vitamin B12, zinc, and iron, which are often not naturally present in plant-based alternatives. To enable fair and meaningful comparisons, the development of equivalence criteria is essential. These criteria will ensure that plant-based alternatives eligible for health-related positioning provide levels of key nutrients comparable to their animal-based counterparts.

The aim is that plant-based products will be assessed against the same criteria as their animal-based counterparts. Products that meet the equivalence criteria are not restricted, and may be assigned levels 1–5, while those that do not meet the equivalence criteria should not obtain a health-related positioning and are therefore classified within levels 3–5. Animal-based products are exempt from these equivalence criteria. Note that hybrid products are qualified as animal-based, if at least 70% of the ingredients are of animal origin. For hybrid products with less than 70% of the ingredients from animal source, the equivalence criteria are applicable as well.

These equivalence criteria consist of nutrient-specific thresholds, focusing on protein and selected micronutrients. Despite their relevance for public health, protein quality cannot be included in the criteria, because the required data about the amino acid composition and digestibility are not available from the ingredient list, back-of-pack nutrition information panel, or from nutritional databases. Micronutrient selection is based on the dietary contribution of animal-based products and their relevance to public health. Threshold values are informed by the nutrient content of animal-based products that qualify for Choices level 1 or 2.

The development process of the equivalence criteria followed five steps:

1. selecting data sources to determine nutrient content and to evaluate the criteria;
2. selecting qualifying nutrients based on the extent to which a serving contributes to the nutrient intake and alignment with nutritional policies;
3. correcting for potential differences in the bioavailability of nutrients in animal- and plant-based foods and diets;
4. defining thresholds and criteria for qualifying nutrients so that, if applied, the majority of animal-based products would comply with these criteria;
5. evaluate the criteria using plant-based products to assess the applicability and discriminatory power.

#### 2.1.1. Defining Data Sources to Determine Nutrient Content in “Healthier” Animal-Based Products

The first step involved compiling a database of cheese, milk, and meat products that qualify as “healthier” (Choices level 1 or 2) using Dutch and Swedish food composition databases [31,32]. All products were selected using the product group classifications in those databases, and then further filtered using the product descriptions. Details of the composition of the database are listed in Table 1. These products were used to assess the average nutrient content. These findings were compared with values reported in the scientific literature to ensure robustness and external validity.

#### 2.1.2. Selecting Qualifying Nutrients

Next, qualifying nutrients were selected based on several factors: the extent to which a typical serving of meat, milk, or cheese contributes to the diet (10% was chosen as a minimum) and existing fortification policies and guidance for plant-based alternatives, issued by national and global health authorities, were taken into account, as well as the broader public health significance of each nutrient.

As reference values for the recommended intake amount per day, European Population Reference Intakes (PRIs) and, when PRIs were not available, adequate intakes (AIs) were considered [33]. To obtain a reference value for the entire population, PRIs for age-groups >18 y of males and (non-pregnant and non-lactating) females were averaged. The median of portion sizes as published in documents of 34 European countries were used to determine a typical portion size for fresh cheese products (60 g), milk products (200 g), and meat products (100 g) [34].

#### 2.1.3. Account for Differences in Bioavailability

Potential differences in the bioavailability of nutrients between animal- and plant-based sources were reviewed and assessed. If evidence indicated meaningful differences, the nutrient thresholds were adjusted to account for lower absorption or utilization of specific nutrients in plant-based alternatives.

#### 2.1.4. Define Nutritional Equivalence Criteria

Nutrient thresholds for determining nutritional equivalence were based on the average nutrient content of selected animal-based products. Assuming nutrient contents are normally and independently distributed, approximately 50% of products will exceed the mean for any single nutrient, and about 25% will exceed the mean for two nutrients simultaneously. The probability that a product’s nutrient content exceeds one standard deviation below the mean is approximately 0.84 (based on standard normal distribution tables). Therefore, the likelihood that a product exceeds this threshold for two independent nutrients is about 0.84 × 0.84 = 71%. By setting the threshold at one standard deviation below the mean, most animal-based products will meet multiple nutrient criteria.

A plant-based alternative is considered nutritionally equivalent if it meets the protein threshold and satisfies at least a subset of the specified micronutrient thresholds. Various combinations of these thresholds were applied to a set of nutritionally “healthier” animal-based products to identify the most stringent combination that still allows compliance by a substantial majority (70–80%) of these reference products.

#### 2.1.5. Evaluate Nutrition Equivalence Criteria

Finally, the developed equivalence criteria were used to evaluate a range of plant-based alternatives to milk and meat products using data from the Dutch and Swedish food composition databases (see Table 1). This step helped to assess the applicability and discriminatory power of the criteria in real-world product categories.

### 2.2. Non-Sugar Sweeteners

NSSs are low- or zero-calorie substances, both artificial and naturally derived, developed as alternatives to sugar. Their use has increased globally in response to recommendations from health authorities, including WHO, to reduce free sugar intake. This trend has raised concerns regarding the safety and potential health implication of NSS consumption, particularly given their relatively recent introduction into the food supply, largely within the past 50 years.

In the current Choices framework, NSSs are not considered a key nutrient, and their presence in product formulations does not influence the health assessment. To evaluate whether and how NSSs should be incorporated into the Choices NPS, recent evidence on the long-term health effects of NSS consumption was reviewed and how this evidence is reflected in public health guidelines was examined. Central to this assessment were several key sources: the WHO’s recent systematic review on the health effects of NSSs [35], its accompanying guideline on NSS use [36], and a systematic review by Mathur and Bakshi [37]. These documents were critically evaluated and discussed by the ISC.

Furthermore, a scoping review following the framework by Arksey and O’Malley [38] was conducted to understand the extent to which information regarding the consumption of NSSs is incorporated in health policies, recommendations, and guidelines. A search strategy was developed on Scopus. An additional search of international and national publications, combined with a snowballing approach, was conducted to identify up-to-date guidelines, policies, FBDGs, and front-of-package labeling systems across selected countries worldwide. The search query and selection process can be found in the Supplementary Materials Table S1 and Figure S1.

In parallel, how NSSs are addressed in other existing nutrient profiling systems was examined to understand their methodological approaches. To assess the potential implications of incorporating NSSs into the Choices algorithm, proposed adaptations to selected indicator foods, including milk products and sugar-sweetened beverages (SSBs), were applied. This modeling exercise enabled the ISC to visualize and assess the impact of different algorithm modification options on product classification. Based on these analyses, the ISC deliberated on whether the inclusion of NSS criteria was warranted and, if so, how such criteria could be operationalized in a scientifically robust and policy-relevant manner.

### 2.3. Trans-Fat

Trans-fatty acids (TFAs) are a type of unsaturated fat originating from both industrial and natural sources. The industrially produced form (iTFA) is commonly found in products such as margarine, vegetable shortening, vanaspati ghee, as well as in fried and baked goods like crackers, biscuits, and pies. Street foods and restaurant-prepared fried items are also frequent sources. Naturally occurring TFAs are present in meat and dairy products derived from ruminant animals, including cows, sheep, and goats. Evidence indicates that both industrial and ruminant-derived TFAs are equally detrimental to health [39]. However, while naturally occurring TFAs are difficult to avoid entirely, iTFAs can be effectively eliminated from the food supply and substituted with healthier fats or oils, without compromising product cost, taste, or availability.

In the current Choices NPS, iTFA is a key nutrient for three product groups: “fats, oils and spreads”, “savory snacks” and “all other products”. The thresholds were last reviewed in 2018 [20] and multiple iTFA levels were not included in the five-level extension, except for “savory snacks” [21]. After publication of this framework, one stakeholder noted that, for some high-fat products, the existing threshold of 0.5 g/100 g may not be technically achievable.

To determine whether alignment with WHO guidance would be appropriate, the ISC first assessed the potential implications of adopting WHO-aligned thresholds. The WHO REPLACE limit of ≤2% iTFAs of total fat [40] was treated as a candidate threshold 4 (T4), distinguishing between levels 4 and 5, and the WHO Europe NPS limit of ≤1% of total fat [26] as threshold 3 (T3), distinguishing between level 3 and 4. Under this system, iTFA criteria are applicable to all food groups, not only to specific food groups; any product with iTFAs ≤ 1% of total fats is eligible for levels 1–5; products with 1% < iTFAs ≤ 2% are eligible for levels 4–5; and products with iTFAs > 2% of total fats are restricted to level 5. These thresholds differ from one another and are fundamentally different from the current Choices criteria, which apply only to three product groups and are expressed as grams per 100 g of product rather than as a percentage of total fat.

To evaluate the impact of adopting WHO-aligned thresholds, the Dutch food composition database (*N* = 2323) [31] was analyzed. Products with TFA > 0 g/100 g (*N* = 536) were selected, and items likely containing only ruminant-derived TFAs were removed based on product descriptions (e.g., bacon, beef, butter, cheese, chicken, cream, dairy, milk, meat, veal, yogurt), leaving *N* = 192 products. The dataset is available in the Supplementary Materials Table S2. Some products may still contain mixed sources of TFAs (e.g., biscuits, croissants); however, due to uncertainty about origin, these were retained. Products were then classified into Choices product groups and assessed using both the current and the proposed iTFA criteria to determine the effect on overall Choices levels.

### 2.4. Whole Grain

Whole grains consist of the intact, ground, cracked, flaked, or otherwise processed kernel after the removal of inedible parts and include all anatomical components, including the endosperm, germ, and bran in the same relative proportions as in the intact kernel [41]. They are widely recognized as part of a healthy diet, with numerous observational studies demonstrating beneficial associations between whole-grain intake and reduced risks of mortality, chronic diseases, and their related risk factors [42,43,44,45]. Accordingly, whole-grain intake is considered an important component of a balanced diet and is recommended within dietary guidelines across the globe [46]. Despite these widespread recommendations, however, whole-grain consumption remains low worldwide [47].

The current Choices criteria do not explicitly account for whole-grain content in assessing food quality but instead rely on fiber content as a proxy measure. Previous research has explored modifications to include whole-grain criteria in other nutrient profiling systems, such as the HSR and Nutri-Score, demonstrating that explicitly considering whole grain allows for better differentiation between high- and low-whole-grain foods [48,49]. This, in turn, promotes whole-grain products and improves alignment with dietary guidelines. To explore the potential benefits and implications of including whole-grain criteria in the Choices NPS, several options were tested.

The most recent recommendations for defining a whole-grain food from the Whole Grain Initiative (WGI) [41] were applied to evaluate potential modifications to the Choices NPS. According to this definition, a food is classified as a whole-grain food if it contains at least 50% whole-grain ingredients by dry weight. Furthermore, the WGI recommends that products containing at least 25% whole-grain ingredients by dry weight may make a front-of-pack claim about the presence of whole grain but cannot use the term “whole grain” in the product name. For the Choices product groups “plain noodles and pasta”, “flavored noodles and pasta”, “bread”, “breakfast cereals” and “grains” under the “sources of complex carbohydrates” category, the whole-grain thresholds applied were defined as shown in Table 2. The current fiber thresholds are included as well.

Three modifications of the Choices criteria were evaluated and compared with the current approach, which uses fiber thresholds only:

- whole-grain and fiber criteria;
- whole-grain instead of fiber criteria;
- whole-grain or fiber criteria.

These modifications were tested using food composition data derived from the Australian Food, Supplement and Nutrient Database (AUSNUT) 2011–13 [50] and the Australian whole-grain database for the whole-grain content of foods [51,52].

## 3. Results

### 3.1. Plant-Based Alternatives to Meat and Dairy

#### 3.1.1. Determining Nutrient Content

The first step to develop equivalence criteria for plant-based alternatives was to determine the nutrient content in “healthier” animal-based products. Table 3 presents the average nutrient content (per 100 g) for selected “healthier” cheese, milk, and meat products, classified as Choices level 1 or 2. A comparison of products from the Dutch and Swedish databases revealed largely similar compositions, and therefore it was reasonable to combine both datasets and analyze the average nutrient content.

Comparison of the database-derived nutrient values with the literature data for cheese [53], milk [54,55,56], and meat [57] products showed that the “healthier” products identified by the Choices criteria fall within expected nutrient ranges. Soft cheeses qualify more often as “healthier” due to lower saturated fat and sodium, though they generally contain fewer micronutrients than hard cheeses. For milk products, average protein values were slightly higher—largely due to high-protein yogurts—while fat-soluble vitamin values were lower, consistent with lower-fat formulations. Iodine levels fell within the broad range reported in the literature. “Healthier” meat products contained similar or higher micronutrient levels than the wider meat category, demonstrating that lower levels of saturated fat and sodium can be achieved without compromising nutrient density. More details are provided in the Supplementary Materials Table S3.

#### 3.1.2. Selecting Qualifying Nutrients

The next step was to select the qualifying nutrients, based on their contribution to reference values (PRIs or AIs) from typical portions of cheese, milk, and meat; their population level intake from these foods; alignment with food-based dietary guidelines; relevant fortification policies; and their overall public health significance. The contribution of a typical portion size of “healthier” cheese, milk, and meat products to the PRIs and AIs are calculated in Table 4. As expected, it was noted that a portion of “healthier” milk and meat products would supply >10% of the PRIs or AIs of many nutrients, such as protein, minerals Ca, Fe, I, P, Se, and Zn and vitamin B2 and B12, which are required on a daily basis. Note that the database does not contain organ meat, which contains much higher amounts of micronutrients. However, the contribution of a 60 g portion of “healthier” cheese products to the PRIs or AIs did only exceed 10% for P (15%).

Comerford et al. [58] reviewed dairy recommendations in food-based dietary guidelines. They found that most nutrient-based dairy messaging relating to underconsumption was focused on Ca, followed by I, K, vitamin D, and protein. In some countries, vitamin D is added to milk. Several countries are encouraging the fortification of plant-based milk and meat alternatives. Musa-Veloso and Juana [59] give a useful overview of the regulation and labeling of plant-based beverages and meat alternatives in Canada and the United States. In the Supplementary Materials, a summary of national requirements or guidelines for the fortification of plant-based milk (Table S4) and meat (Table S5) products is included. For milk alternatives, Ca, Vitamin D, and B12 are often mentioned. For meat alternatives, it seems that less regulation and guidance is available. However, it was found that the majority (85/104 products) of meat alternative products found in the Dutch and Swedish databases have an iron content ≥1.0 mg/100 g and a minority (33/104 products) had a vitamin B12 content > 0 µg/100 g, suggesting that these products have been fortified [31,32].

The question was whether the nutrients provided by cheese, milk and meat products are nutrients of global public health concern [60]. Phosphorus is not a health problem. Protein, Ca, Fe, I, Mg, Se, Zn, B2, and B12 are nutrients that are globally relevant for public health and deficiencies may be endemic in populations that exists on diets lacking dairy products and meat. Vit D is relevant for public health but only present in cheese or milk when fortified (national legislation). In conclusion, it was agreed to use the following nutrients as qualifying nutrients:

- for cheese products: none;
- for milk products: protein quantity, Ca, B2, B12;
- for meat products: protein quantity, Fe, Zn, B2, B12;

If countries would use the Choices International criteria as a reference for their national NPS, they may consider to include I, Se, Zn, vitamin D, and/or other micronutrients depending on the public health concerns and/or fortification requirements for milk products.

#### 3.1.3. Accounting for Bioavailability Differences

The following step was to account for differences in the bioavailability of qualifying nutrients in animal-and plant-based products or diets.

1. Protein

Although protein quality and digestibility may differ between animal-source foods and their plant-based alternatives, there are practical limitations to accounting for these differences. Plant-based alternatives are produced using a wide variety of protein sources, and food labels typically do not provide quantitative information on the specific sources used. It was decided not to correct for protein quality or digestibility.

2. Calcium

The bioavailability of calcium in plant-based beverages varies depending on Ca salt and matrix. Heany et al. [61] reported a bioavailability (in comparison to cow’s milk) of 75% for Ca_3_(PO_4_)_2_. Zhao [62] reported a bioavailability of 83% for Ca_3_(PO_4_)_2_ but ~100% for CaCO_3_. Biscotti et al. [63] mentioned that myo-inositol phosphates, phytate, and oxalate may interfere, as well as other vitamins and minerals, and that sedimentation of Ca salts may limit the intake. It seems that it is possible, with optimal Ca fortification, that plant-based beverages may provide an equivalent source of Ca as cow’s milk, but, to be on the safe side, it was decided to correct for bioavailability with a factor 1/75% = 1.3.

3. Iron

Heme iron from hemoglobin and myoglobin is abundant in meat and is also highly bioavailable and readily absorbed, since it is uninhibited when digested. Beef is, on average, the highest heme iron-rich source, with an average of approximately 58% of its total Fe content being heme iron [57]. Non-heme iron has a bioavailability of 2–20%. Heme iron is two to three times more bioavailable than non-heme iron [64]. Taking an average bioavailability of 10% for non-heme iron and 30% for heme iron, the bioavailability of iron in meat was calculated: 58% × 30% + 42% × 10% = 22%. In comparison with the bioavailability in plant-based products (fortified with 100% non-heme iron) of 10%, this resulted in a correction factor of 22%/10% = 2.2.

4. Zinc

For meat alternatives, the European Food Safety Authority (EFSA) published a report [65] regarding the average daily required intake of Zn depending on phytate intake and body weight. Plant-based diets have a roughly three-fold higher phytate intake than their nonvegetarian counterparts [66]. The EFSA study found that the average body weight Zn requirement at a phytate consumption of 1200 mg/day is 12.2 mg/day compared to the 7.25 mg/day at 300 mg of phytate per day. This resulted in a correction factor of 12.2/7.25 = 1.7.

5. B2

Bioavailability in animal-based foods is 61% and in plant-based foods it is 65% [67]. However, further studies are needed to make meaningful comparisons. Therefore, it was decided not to correct for relative bioavailability, correction factor = 1.

6. B12

The absorption of vitamin B12 is limited and becomes dose-dependent due to saturation effects [68,69]. At daily intakes of 4–7 µg/day, intestinal absorption plateaus, with maximum uptake reaching 1.5–3 µg per meal. The bioavailability of vitamin B12 from animal-sourced foods, such as meat and milk, is consistently high—approximately 65% [67,70]. In contrast, the bioavailability from fortified plant-based foods, such as bread and rice, is lower, averaging around 50% [71,72]. This resulted in a correction factor of 65%/50% = 1.3.

#### 3.1.4. Determining Nutrient Thresholds

Next, the equivalence thresholds for qualifying nutrients were determined based on the mean nutrient content minus the standard deviation of “healthier” (Choices level 1 or 2) milk and processed meat products (see Table 5). B12 content in meat products exhibits considerable variability. To determine threshold values, generally the average minus the standard deviation was used. However, for B12 in meat products, a threshold of 70% of the average was instead applied, as the average and standard deviation were almost equal. The % of “healthier” products that had a nutrient content greater than the threshold value varied between 81 and 94%, which is consistent with the 84% probability based on normal distributions. The only exception is for B12 in processed meat products, as it was chosen to calculate the threshold differently.

The correction factors for bioavailability were then applied, as discussed in the previous section. After multiplying the threshold values for animal-based products with these correction factors and rounding, the threshold values for plant-based products were determined and are included in Table 5.

#### 3.1.5. Defining and Evaluating Equivalence Criteria

The final step in the development process was to define and evaluate the equivalence criteria for plant-based products. As outlined earlier, these products are assessed against the same nutrient criteria as their animal-based counterparts. Four alternative approaches for setting these equivalence criteria were evaluated, and the proportion of animal-based products unaffected by each option was calculated (Table 6). Only 75% of milk products and 48% of meat products met all qualifying nutrient thresholds, indicating that a fully strict approach would exclude too many products. Option 2 was selected—the strictest approach that would still allow >70% of the “healthier” animal-based products to remain unaffected if equivalence criteria were applied. Under this approach, a plant-based alternative is considered nutritionally equivalent if it meets the protein threshold and all but one of the qualifying micronutrient thresholds.

Now that we had defined the equivalence criteria, the new system was applied to a range of plant-based milk and meat alternatives. If a “healthier” plant-based product complies with the equivalence criteria, its Choices level remains unaffected. “Healthier” products that do not comply with the equivalence criteria are downgraded to level 3 and lose their health-related positioning. The results are listed in Table 6. Only 40% of plant-based milk alternatives and 4% of plant-based meat alternatives meet the established equivalence criteria.

These findings demonstrate that the equivalence criteria effectively distinguish between plant-based alternatives that are fortified to provide micronutrients typically supplied by animal-based products and those that are not sufficiently fortified. Moreover, the results indicate that only a small proportion of current plant-based alternatives deliver protein and micronutrient levels comparable to their animal-based counterparts, underscoring the need for clearer nutritional standards for plant-based alternatives within healthy and sustainable diets.

### 3.2. Non-Sugar Sweeteners

#### 3.2.1. Understanding Long-Term Health Associations with NSS Consumption

The new WHO guideline on NSSs [36] advises against their use for body weight control or reducing the risk of non-communicable diseases. This recommendation is based on the systematic research review conducted by Rios-Leyvraz and Montez [35], which concluded that replacing sugar sweeteners with NSSs did not promote long-term weight loss in adults and children. However, their meta-analysis of 29 randomized control trials (RCTs), found that greater NSS consumption, compared with free sugars, was associated with a mean body weight reduction of 0.71 kg. Additional studies have supported these findings, concluding that replacing free sugars with NSSs can reduce total energy intake, creating a calorie deficit and short-term weight loss [73].

However, cohort/case–control studies suggest an increased risk of incident obesity (HR 1.76) and small increase in body mass index (BMI) (+0.14 kg/m^2^) associated with NSS consumption. Many studies assess energy intake by comparing NSSs with nutritive sweeteners or sugar rather than with no-sugar comparators (e.g., water, placebo)—an important limitation that could lead to different results [74]. According to Rios-Leyvraz and Montez [35], when NSSs are used specifically as replacements for sugars (mostly in the form of replacing SSBs with NSS-sweetened beverages), the effects on body weight and BMI are small, and not statistically significant (moderate certainty evidence).

Overall, findings are inconsistent as to whether NSSs lead to weight loss or weight gain [37,75]. Mathur and Bakshi [37] reported that NSS exposure did not conclusively induce increased food intake or a change in subjective appetite ratings. Appetite biomarkers like ghrelin, gastric inhibitory peptide, C-peptide levels, and Peptide YY remained mostly unaffected by NSSs. Meta-analyses of human randomized control studies showed a reduced energy intake and body weight. No significant change was seen in blood glucose levels or post-prandial glycemic or insulin response after the consumption of NSSs. In the systematic review by Rios-Leyvraz and Montez [35], cohort/case–control studies yielded mixed results for most health outcomes, underscoring the need for further research to clarify potential long-term risks or benefits. Evidence regarding effects on gut health at human-relevant doses remains inconclusive [37].

Certain subpopulations warrant specific attention. For pregnant women, higher NSS intake has been associated with increased risk of preterm birth (low-certainty evidence) and possible adiposity in offspring (very low-certainty evidence) [35]. Data for children are limited and largely inconclusive.

In summary, most studies, which are prospective cohort, observational, and cross-sectional studies, suggest that NSS use may promote obesity and metabolic syndrome in adults, but these findings are prone to confounding variables and reverse causation. Mechanistic evidence is mostly based on in vitro and in vivo animal studies. The same causal pathways may not be operative or relevant in humans. We did not encounter adverse effects for Stevia. Most studies have looked at Aspartame, Acesulfame K, Sucralose, Saccharin, and others. There was not much evidence against polyols either (only the laxative effect cited as a problem) except against erythritol. Some formulations of Stevia have erythritol. Whilst the short-term effects of the replacement of free sugars by NSSs may make a positive health impact, the evidence on the long-term health effects associated with NSSs are inconclusive.

#### 3.2.2. Policies and Regulations Related to NSSs

The WHO has developed a manual on SSB taxation policies [76], recommending that member states consider taxing beverages containing NSSs in addition to those sweetened with sugar. UNICEF also supports taxation of both sugar-sweetened and artificially sweetened beverages as a means to reduce children’s consumption of sugars and SSBs [77].

Policies and recommendations related to NSSs are summarized in Table 7. While global policies have recently been updated to generally restrict NSS consumption, relatively few national policies specifically address NSS use.

#### 3.2.3. Inclusion of Non-Sugar Sweeteners in the Choices NPS

The Choices ISC agreed to include NSSs in the criteria for three reasons: (1) potential, albeit weak, evidence of adverse health effects; (2) the goal of fostering a less sweet taste profile; and (3) alignment with global and national policies. NSSs are defined according to the WHO as all synthetic and naturally occurring or modified non-nutritive sweeteners not classified as sugars; sugar alcohols and low-calorie sugars are excluded from this definition. This definition also covers naturally occurring sweeteners such as Stevia and its derivatives. Despite the lack of evidence of adverse health effects of Stevia (derivatives), the ISC decided to include these to encourage products with a less sweet taste profile and to align as much as possible with global policies.

Two approaches were considered:

1. Option 1 (strictest): Products containing NSSs are limited to Choices levels 3–5. Basic food products with NSSs could not be positioned as “healthier” (levels 1–2), and discretionary foods (e.g., SSBs) would default to the lowest levels (4–5 in a 5-level FOPNL).
2. Option 2 (less strict): Products containing NSSs are downgraded by one level compared to identical products without NSSs (except those already at level 5).

Both approaches were tested on selected milk products and beverages (Supplementary Materials Tables S6 and S7). The modeling showed that both approaches encourage sugar reduction and reformulation toward less sweet taste profiles by differentiating products based on NSS content. Considering the weak evidence of adverse health effects, the ISC adopted the less restrictive Option 2, whereby products containing NSSs are downgraded by one level compared with identical products without NSSs. This permits basic foods with NSSs to remain eligible for level 2 and thus a “healthier” related positioning, but not for a positive logo, which is restricted to level 1 products.

### 3.3. Trans-Fat

The feasibility and implications for overall Choices level of aligning the Choices iTFA thresholds with WHO recommendations were analyzed by assessing the Choices levels of products with iTFAs > 0 from the Dutch food composition database. The number of products classified under the current criteria and under the proposed WHO-aligned criteria is shown in Table 8. For most products, the different iTFA criteria do not affect the Choices level; for example, 10 of the 11 products with current Choices level 1 would also be classified as 1 under the WHO-aligned iTFA criteria. Some products, mainly from the product groups “main meals” and “sweet snacks”, are downgraded to level 4 or 5, exceeding the ≤1% and ≤2% iTFAs of total fat thresholds. For these groups, the proposed criteria were generally stricter, although the source of TFAs was not always clear. In contrast, for “oils, fats and spreads” and “savory snacks”—product groups currently subject to iTFA limits—the proposed criteria generally assigned less restrictive levels than the current system. While 64 products are classified as level 5 under the current criteria, 19 of these would fall into levels 1–4 under the proposed criteria. A notable example is linseed oil, which contains 1.0 g iTFAs per 100 g product—exceeding the current threshold of 0.5 g per 100 g product, but meeting the proposed threshold of ≤1 g per 100 g total fat. Under the current system, linseed oil would be assigned level 5; under the proposed system, it would be assigned level 1.

It was also noted that, if all oils and fats used as food ingredients complied with the revised iTFA thresholds, then products containing these ingredients (e.g., margarines, snacks, main meals) would also be compliant. Thus, in the absence of iTFA data on food labels, one may make reasonable assumptions about the iTFA levels depending on the iTFA elimination status of fats and oils in a given country. The proposed criteria also address feedback that the current threshold of 0.5 g per 100 g product may not be technically achievable for products high in fats or oils. Based on this impact assessment with the clear policy direction provided by WHO REPLACE and the WHO Europe NPS, the ISC concluded that adopting these WHO-aligned thresholds was feasible and appropriate, and therefore adopted the revised iTFA criteria.

### 3.4. Whole Grain

Following relevant exclusions and assumptions, 483 foods from AUSNUT 2011-13 [50] were included and classified as “sources of complex carbohydrates”.

**Figure 1:**
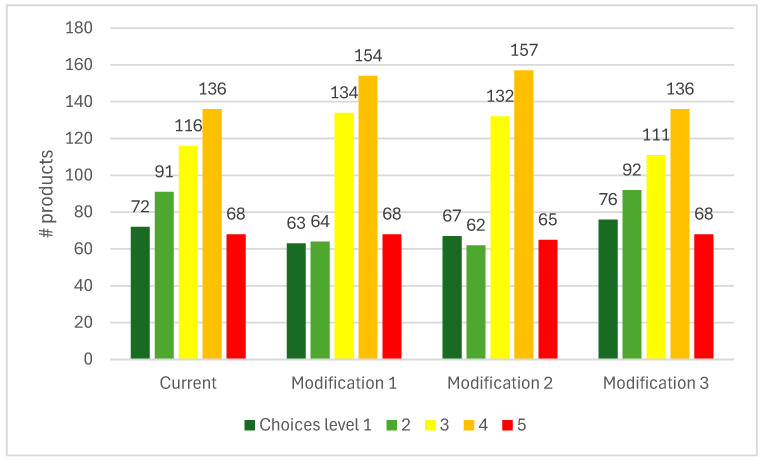
Number of products assigned Choices levels 1–5 of all sources of complex carbohydrate foods (*N* = 483) in AUSNUT 2011-13 [50] across the current Choices criteria (fiber criteria only) and modifications 1 (whole-grain and fiber criteria); 2 (whole-grain instead of fiber criteria); and 3 (whole-grain or fiber criteria).

Applying the current Choices criteria saw the highest number of foods scoring level 4, followed by levels 3, 2, 1, and then 5 (Figure 1). When applying the whole-grain criteria in combination with the fiber criteria (modification 1) or instead of the fiber criteria (modification 2), there were small decreases in foods scoring level 1, larger decreases in foods scoring level 2, and increases in foods scoring level 3 and 4. This indicates a proportion of foods, including some that currently score levels 1 or 2, contain <25% whole grain. By contrast, applying the whole-grain *or* fiber criteria (modification 3) resulted in minimal changes, with only a few rice-based products, including brown and red rice, being affected. Whole-grain rice is known to have relatively lower fiber levels compared with other whole-grain products. Based on the validation with indicator foods, using brown rice as one of the indicator foods for the “grains” product group, the fiber criteria were adjusted such that brown rice would score level 2 and white rice level 3 [21].

Overall, modifications to the Choices criteria to include whole-grain thresholds improved differentiation between whole- and refined-grain foods, better promoting products with high whole-grain content. These findings are consistent with research on modifying the Nutri-Score nutrient profiling system, which also showed that foods with greater whole-grain content scored more favorably, particularly within grain-specific food groups [48]. Applying criteria based on either whole grain or fiber, selecting whichever resulted in a better score, did not improve differentiation between whole- and refined-grain foods. Only a small number of rice-based products (*n* = 5) that were high in whole grain but low in fiber received improved scores.

In contrast, modifications 1 and 2 resulted in a modest but significant shift, moving some complex carbohydrate products with previously healthier assessments (levels 1 and 2) to lower nutrient quality levels 3 and 4. Importantly, the fiber thresholds used in the current Choices NPS still classified the majority of complex carbohydrate foods in the same or similar quality levels as when whole-grain content was included.

Finally, there are practical limitations to including whole-grain criteria. Data on whole-grain content are generally not available on food labels, limiting their applicability. Combined with the unintended downgrading of otherwise high-quality foods, these considerations led us to decide not to include whole-grain criteria in the Choices International five-level criteria at this time.

Due to practical considerations, we chose to define dietary fiber as simply the total amount included on the nutrition fact panel or equivalent on the product label. This is with the recognition that country regulatory agencies deal with the definition of dietary fiber differently, many adopting the WHO 2009 definition [89], which includes intrinsic fibers and extrinsic (i.e., added) fibers that confer physiological benefits. Some other countries use older, broader definitions of dietary fiber that includes all intrinsic and extrinsic fibers. Also, countries differ in their acceptance of the degree of polymerization 3–9 indigestible carbohydrates as dietary fiber. Nutrient scoring systems, in general, must use total dietary fiber values that are found on product labels.

### 3.5. The Choices 2025 Criteria

In summary, the review resulted in the following updates to the Choices NPS compared with the criteria published in 2021 [21]:

- Plant-based alternatives to meat and milk products are now assessed against the same criteria as their animal-based counterparts. These products are eligible for level 1 or 2 only if they meet the established equivalence criteria. The separate food group “non-dairy milk alternatives” has therefore become redundant. For plant-based alternatives to other foods groups (e.g., cheese, fish, eggs, etc.), no specific equivalence criteria have yet been defined, but such products should be assessed using the same criteria as their animal-based equivalents.
- Products containing non-sugar sweeteners (NSSs) are downgraded by one level compared with identical products without NSSs (except those already classified at level 5).
- For industrially produced trans-fatty acids (iTFAs), products with iTFAs ≤ 1% of total fats are eligible for levels 1–5; those with 1% < iTFAs ≤ 2% are eligible for levels 4–5; and products with iTFAs > 2% of total fats are restricted to level 5. These criteria are applicable to all product groups.

The updated Choices NPS is given in Table 9.

## 4. Discussion

This study describes the 2025 revision of the Choices NPS and the scientific rationale for its updates. The revision process was guided by recent evidence, international policy developments, and stakeholder feedback. The main outcomes include the following: (1) the adoption of equivalence criteria for plant-based alternatives to meat and dairy products; (2) the inclusion of NSSs as a factor that lowers a product’s classification by one level and makes it ineligible for a positive logo; (3) the alignment of iTFA thresholds with WHO guidelines; and (4) a review of whole-grain criteria, which were ultimately not adopted. Together, these updates strengthen the scientific coherence, policy relevance, and international applicability of the Choices NPS.

### 4.1. Plant-Based Alternatives

Animal-based products are classified as basic food groups because they provide essential nutrients to the diet. The equivalence criteria ensure that plant-based alternatives can obtain a health-related positioning only if they provide comparable nutritional value. These criteria enhance fairness and comparability across animal- and plant-based products. Nutrient adequacy (protein quantity and key micronutrients) is now comparable, although labeling constraints limit the assessment of protein quality and digestibility. Some gaps remain (e.g., for fish and eggs) and equivalence criteria were defined using European data. Using the same methodology, countries may adapt the equivalence criteria to their local nutritional needs, food habits, and composition data. International and national policies provide limited—and, where available, mainly voluntary—guidance on the nutritional requirements for plant-based alternatives to animal-based foods. This is illustrated by the fact that only 40% and 4% of plant-based milk and meat alternatives complied with the equivalence criteria. To ensure that plant-based alternatives to animal-based foods are nutritionally equivalent, mandatory guidance is required on their nutritional composition.

### 4.2. Non-Sugar Sweeteners

While the short-term effects of the replacement of free sugars by NSSs may have positive health effects, evidence on the long-term health impacts remain inconclusive. The revised NPS lowers the classification of a product containing NSSs by one level to reduce sweet-tasting products and further align with WHO guidelines. Product reformulation efforts to reduce both sugar content and product sweetness should be supported by FOPNL, regulatory measures, and fiscal and marketing policies. The revised Choices NPS can support these policies more coherently than before.

### 4.3. Industrial Trans-Fatty Acids

The revised iTFA criteria are now fully aligned with WHO Europe marketing restrictions and WHO REPLACE targets, setting thresholds of ≤1% and ≤2% of total fats for classification into levels 1–5 and 4–5, respectively. This represents a major improvement in harmonization with international standards while maintaining feasibility for manufacturers. In markets where industrial trans-fats have been eliminated from the food supply, this change primarily enhances regulatory consistency rather than imposing new reformulation demands. In other markets, it is important that the Choices NPS, when used to support FOPNL or other nutritional policies, aligns with global iTFA elimination targets.

### 4.4. Whole-Grain

The potential inclusion of whole-grain thresholds was explored but not adopted in the 2025 revision. Testing showed somewhat better differentiation between refined and whole-grain foods when whole-grain criteria were applied, either alongside or instead of fiber thresholds. However, many otherwise high-quality products were downgraded and practical constraints—such as data availability and labeling information—outweighed marginal improvements in classification. As whole grains provide health benefits beyond fiber, future inclusion should be reconsidered once compositional data and labeling standards improve.

### 4.5. Comparison with Other Nutrient Profiling Systems

Other NPSs, such as Nutri-Score, HSR, and the Nutrient Rich Food (NRF) Index [24,25,90] do not distinguish between basic and discretionary food groups and do not use equivalence criteria to assess plant-based alternatives. Each system assesses animal- and plant-based products using the same criteria, although Nutri-Score assigns penalty points specifically for red meat. Neither Nutri-Score nor HSR considers micronutrients as qualifying components, whereas the NRF does. A recent study [91] assessed the nutritional quality of beverages in Sweden using the Nutri-Score and NRF and compared these with Keyhole eligibility. Key nutrient quality attributes in beverages are calorie, sugar, and fat content, the presence of non-sugar sweeteners, and the overall nutrient density, which are captured to varying degrees by the three indicators. Notably, the Keyhole does not specify minimum protein or micronutrient levels for plant-based milk alternatives. Among all milk- and plant-based beverages, only 20% of products were classified similarly by the Keyhole, Nutri-Score, and NRF systems. This example highlights the challenge of aligning NPSs with national FBDGs.

Despite the remarkable progress since the WHO called for the global elimination of iTFAs in 2018, WHO-recommended policies covered only 46% of the world’s population in 2023 [92]. Therefore, it is important that NPSs serving as a global reference for national systems—such as the Choices NPS—remain aligned with WHO iTFA elimination policies. Notably, Nutri-Score, HSR, and NRF do not consider iTFA a disqualifying nutrient.

### 4.6. Strengths and Limitations

A key strength of this revision is its evidence-based and consultative process. Multiple ISC-led studies, involving international students and experts, informed the decisions, ensuring scientific credibility and global relevance. The use of indicator foods and comparison with national FBDGs helped validate the system against real-world dietary standards.

Limitations include the restricted geographic coverage of available food composition databases. Although the used databases are not globally representative, their use is justified since the composition of “healthier” meat and milk products is likely similar across countries. Furthermore, whole-grain foods across North America, Europe, the UK, and Australia/New Zealand are similar [93]. While it was concluded that equivalence criteria were not needed for plant-based cheese alternatives, other product groups were not considered due to incomplete data for some emerging categories (e.g., fish and egg alternatives). These might be included in future reviews as markets for such products expand. Furthermore, the equivalence criteria rely on estimated differences in nutrient bioavailability rather than direct experimental data from well-designed studies on plant-based foods and diets.

### 4.7. Policy and Research Implications

These revisions strengthen the scientific foundation and policy relevance of the Choices system. The Choices system is now well-aligned with WHO policies and has strong potential for adoption in national FOPNL programs and other nutrition policies, including marketing restrictions, procurement standards, and fiscal tools. Future research should focus on incorporating micronutrient density, bioavailability adjustments, and global validation studies to further refine and enhance the system.

## 5. Conclusions

The 2025 revision of the Choices NPS builds on a strong scientific foundation while improving alignment with WHO recommendations and contemporary food system challenges. By refining plant-based equivalence, addressing NSS and iTFAs, and assessing whole-grain inclusion, the updated system enhances both the scientific integrity and policy utility of the Choices criteria. These revisions support the continued use of the Choices framework as a global standard to encourage healthier, more sustainable food environments.

## Figures and Tables

**Table 1:** Characteristics of the database of animal-based and plant-based products from the Dutch [31] and Swedish [32] food composition databases. “Healthier” means Choices level 1 or 2.

Choices Product Group	Database	Product Groups in Dutch and Swedish Databases and Selection Criteria	*N* Total	*n* “Healthier”	% “Healthier”
Cheese products	Combined		114	9	8
	Dutch	Cheese products (73), excluded one hard cheese product from the “healthier” subset ^1^	73	3	4
	Swedish	Toppings (84) (descriptions translated to English and selected only cheese products) excluded one hard cheese product from the “healthier” subset ^1^	41	6	15
Milk products	Combined		183	48	26
	Dutch	Milk products (132) (eliminated human milk and dried milk products)	129	34	26
	Swedish	Milk products (73) (descriptions translated to English and eliminated human milk, dried milk products, and plant-based dairy alternatives)	54	14	26
Processed meat and meat products	Combined		511	298	58
	Dutch	Meat and poultry (216), Cold meat cuts (65), excluded products containing liver, kidney, tongue, or brain	250	139	56
	Swedish	Chicken/bird (44), Meat (174), Sausage (43)	261	159	61
Plant-based milk alternatives	Combined		42	30	71
	Dutch	Dairy and meat alternatives (62) (manually selected milk alternatives)	28	19	68
	Swedish	Milk products (73) (descriptions translated to English and selected plant-based dairy alternatives)	14	11	26
Plant-based meat alternatives	Combined		104	70	67
	Dutch	Dairy and meat alternatives (62) (manually selected meat alternatives)	32	23	72
	Swedish	Quorn, soy protein, and vegetarian products (72)	72	47	65

^1^ These hard cheeses are not mainstream and may differ significantly in palatability and composition from the rest, potentially skewing average values.

**Table 2:** Whole-grain and fiber thresholds for different Choices levels (L1-L4) per product group.

Choices Food Groups	Whole Grain % (Dry Weight)				Fiber Threshold (g/100 g)			
	L1	L2	L3	L4	L1	L2	L3	L4
Plain noodles and pasta, flavored noodles and pasta, breads, breakfast cereals	50	25			6.0	2.8	1.0	0.5
Grains	100	50	25		6.0	2.8	1.0	0.5

**Table 3:** Average content per 100 g of selected nutrients of “healthier” (Choices level 1 or 2) cheese, milk, and meat products from the Dutch and Swedish food composition databases.

Choices Product Group													Vitamin			
	SAFA	Sugar	Na	Protein	Ca	Fe	I	K	Mg	P	Se	Zn	A	D	B2	B12
	g	g	mg	g	mg	mg	µg	mg	mg	mg	µg	mg	RE µg	µg	mg	µg
Cheese products	4.1	3.9	268	9.7	100	0.2	8	105	8	138	3	0.4	86	0.08	0.18	0.39
Dutch FCDB	4.6	4.5	307	7.0	119	0.4	4	93	9	119	0	0.5	119	0.10	0.17	0.39
Swedish FCDB	3.8	3.6	248	11.1	91	0.1	10	111	8	147	4	0.4	70	0.07	0.18	0.39
Milk products	0.4	4.3	44	4.7	119	0.1	14	139	11	99	1.4	0.5	9	0.24	0.16	0.33
Dutch FCDB	0.4	4.3	44	4.4	122	0.1	14	136	11	94	1.4	0.4	10	0.12	0.16	0.31
Swedish FCDB	0.4	4.3	43	5.6	111	0.0	13	146	11	112	1.2	0.5	6	0.55	0.16	0.38
Processed meat and meat products	2.3	0.1	138	23.0	11	1.9	6	359	26	218	11.0	3.3	20	0.43	0.22	1.42
Dutch FCDB	2.4	0.2	123	24.2	11	1.6	4	417	26	227	11.6	3.4	23	0.47	0.17	1.58
Swedish FCDB	2.2	0.1	150	22.0	11	2.2	8	308	26	210	10.5	3.1	18	0.39	0.26	1.28

**Table 4:** Averaged (males and (non-pregnant and non-lactating) females >18 y) PRIs or AIs (used as reference values for the recommended intake amount per day) [33] and the average contribution of a typical portion of “healthier” cheese, milk, and meat products to these reference values.

										Vitamin			
	Protein	Ca	Fe	I ^1^	K ^1^	Mg ^1^	P ^1^	Se ^1^	Zn	A	D ^1^	B2	B12 ^1^
	g	mg	mg	µg	mg	mg	mg	µg	mg	RE µg	µg	mg	µg
PRIs or AIs	58 ^2^	1000	13.5	150	3500	325	550	70	11.5	700	15	1.6	4
Contribution to PRIs or AIs	%												
60 g cheese products	10	6	1	3	2	1	15	3	2	7	0	7	6
200 g milk products	16	24	1	18	8	7	36	4	8	2	3	20	16
100 g meat products	40	1	14	4	10	8	40	16	28	3	3	13	35

^1^ For I, K, Mg, P, Se, Vitamin D, and Vitamin B12 PRIs are not available; AIs were used instead. ^2^ The PRI for protein is 0.83 g/kg. To obtain an estimate of required protein quantity, an average body weight of 64 kg for women and 74 kg for men is assumed [33].

**Table 5:** Development and evaluation of the threshold values for plant-based products.

	Protein	Ca	Fe	Zn	B2	B12
	g	mg	mg	mg	mg	µg
Mean content (and standard deviation) of qualifying nutrients in “healthier” products						
Milk products	4.7 (2.9)	119 (39)			0.159 (0.042)	0.329 (0.163)
Processed meat and meat products	23.0 (4.3)		1.9 (1.2)	3.3 (1.8)	0.216 (0.106)	1.417 (1.411)
Threshold values for animal-based products calculation method: Mean–standard deviation and rounded (B12: threshold = average × 70% and rounded)						
Milk products	2	80			0.1	0.2
Processed meat and meat products	19		0.7	1.5	0.1	1.0
Bioavailability correction factor for plant-based products						
	1	1.3	2.2	1.7	1	1.3
Threshold values for plant-based products						
Plant-based milk alternatives	2	100			0.1	0.3
Plant-based meat alternatives	19		1.5	2.6	0.1	1.3
% of “healthier” products that had a nutrient content > threshold value						
Milk products	90	88			94	81
Processed meat and meat products	90		91	85	93	55
Plant-based milk alternatives	50	63			57	67
Plant-based meat alternatives	24		74	19	49	0

The development process starts with the mean (and standard deviation) content per 100 g of qualifying nutrients of “healthier” (Choices level 1 or 2) milk and meat products from the Dutch and Swedish food composition databases. The nutrient threshold values for animal-based products are derived and multiplied with correction factors to account for differences in bioavailability, resulting in the threshold values for plant-based products. The % of “healthier” products that has a nutrient content greater than the threshold value is calculated.

**Table 6:** The percentage of “healthier” (Choices level 1 or 2) products that would not be affected by various combinations of additional nutritional equivalence criteria.

Nutritional Equivalence Criteria				
Option	1	2	3	4
Milk products	75%	90%	90%	90%
Processed meat and meat products	48%	74%	87%	89%
Plant-based milk alternatives	20%	40%	40%	50%
Plant-based meat alternatives	0%	4%	11%	25%

Option 1: protein and all qualifying micronutrients; 2: protein and all but one qualifying micronutrients; 3: protein and all but two qualifying micronutrients; 4: protein and all but three qualifying micronutrients.

**Table 7:** Summary of polices and recommendations related to NSS intake, by global region.

Geographical Region	Summary of Findings
Global	WHO has developed an NPS and taxation policy strategies applicable across regions [26,76]. UNICEF recommends improvements to FBDGs and child nutrition policies [77].
Africa	FBDGs for South Africa [78], Ethiopia [79], and Kenya [80] do not address NSSs. WHO Africa’s NPS prohibits marketing of NSS-containing products [81].
Americas	Pan American Health Organization (PAHO)’s NPS includes NSS-containing products in regulatory, restriction, and taxation policies [82]. Chile taxes SSBs, including NSS-sweetened beverages [83]. Some states in the USA restrict sweetened beverages in schools and marketing to children [84].
Europe	WHO Europe’s NPS restricts marketing to or being offered to children in schools of NSS-containing products [26]. The Nordic Keyhole label excludes products with NSSs [85]. Nutri-Score penalizes NSSs in scoring [25].
Oceania	NSSs are not explicitly addressed in the Australian and New Zealand Health Star Rating system. Suggestions include clearer FBDG guidance on sweeteners to prevent reformulation toward NSSs [86].
Southeast Asia	In Singapore, products with NSSs can carry the Healthier Choice Symbol but are not allowed in schools [87]. Malaysia’s SSB tax does not apply to NSSs [88].

**Table 8:** Number of products with iTFAs > 0 (*N* = 192) from the Dutch food composition database, classified by current Choices levels and by proposed levels after replacing the existing iTFA criteria with the WHO-aligned criteria.

		Proposed Choices Level					
		1	2	3	4	5	Grand Total
current Choices level	1	10			1		11
	2		28		12	3	43
	3			25	10	2	37
	4				31	6	37
	5	3	3	4	9	45	64
	Grand Total	13	31	29	63	56	192

This table should be read as follows: Of the 11 products with current Choices level 1, 10 would also be classified as level 1 under the WHO-aligned iTFA criteria, whereas 1 would be assigned level 4. Similarly for current Choices levels 2–5.

**Table 9:** Choices five-level criteria for basic and non-basic food groups after the review process. To be assigned to a certain level, product nutrient content should be ≤ (for fiber ≥) than listed thresholds for all nutrients. Products not complying with one of the thresholds for L4 are assigned L5. All products containing iTFAs ≥ 1 g/100 g on total fat are restricted to L4 or L5, and all products containing iTFAs ≥ 2 g/100 g on total fat are assigned L5. All products containing non-sugar sweeteners (NSS) are downgraded by one level. When nutrient thresholds are not provided, they are considered non-critical for that food group. L1–L5 = Choices level 1–5. Unless stated otherwise, nutrient definitions were adopted from the original Choices criteria [13]. SAFA denotes saturated fatty acids; sugar refers to total sugars; fiber represents the total amount declared on the nutrition facts panel or equivalent labeling; and iTFAs denotes industrially produced trans-fatty acids.

	Food Group	L1					L2					L3					L4				
		SAFA	Sodium	Sugar	Fiber	Energy	SAFA	Sodium	Sugar	Fiber	Energy	SAFA	Sodium	Sugar	Fiber	Energy	SAFA	Sodium	Sugar	Fiber	Energy
		g/100 g				kcal/100 g	g/100 g				kcal/100 g	g/100 g				kcal/100 g	g/100 g				kcal/100 g
**Basic food groups**																					
**Fruits and vegetables**	Fresh fruits and vegetables	All compliant																			
	Processed vegetables		0.10	7.0	1			0.25	8.5	0.9			0.40	10.0	0.8			0.65	11.0	0.7	
	Processed fruit	1.1		11.5	1		2		12.5	0.9		3		14.0	0.8		4		19.0	0.7	
	Processed beans and legumes		0.20	5.7	3.5			0.33	7.5	3.2			0.40	10.0	1.7			0.43	10.5	1.1	
**Water**	Plain water, tea and coffee		0.02					0.02													
**Nuts and seeds**	Nuts and seeds	10.0	0.10	7.5			16.0	0.43	14.0			18.0	0.55	30.0			20.0	0.73	36.0		
**Sources of complex carbohydrates**	Plain tubers used as staple	All compliant																			
	Processed tubers used as staple	1.1	0.10	3.0	2.7		3.0	0.35	6.5	2.2		4.0	0.40	10.0	1.5		8.0	1.60	12.0	0.8	
	Plain noodles and pasta		0.10	4.0	6.0			0.20	4.2	2.8			0.48	5.0	1.0			0.80	6.0	0.5	
	Flavored noodles and pasta	2.0	0.50	4.0	6.0		3.5	0.93	4.2	2.8		6.5	1.20	5.0	1.0		8.0	1.50	6.0	0.5	
	Grains	1.2	0.10	4.5	6.0		1.5	0.23	6.0	2.8		1.8	0.48	10.0	1.0		4.0	1.40	12.0	0.5	
	Bread	1.1	0.32	6.0	6.0		1.8	0.40	6.5	2.8		3.5	0.48	9.0	1.0		6.0	0.85	15.0	0.5	
	Breakfast cereals	3.0	0.40	10.0	6.0		3.2	0.50	14.0	2.8		3.3	0.64	15.0	1.0		4.2	0.68	26.0	0.5	
**Meat and alternatives, fish, poultry, eggs**	Unprocessed meat, poultry, eggs	3.2	0.15				3.7	0.17				5.3	0.40				7.5	0.68			
	Processed meat and meat products and plant-based meat alternatives ^1^	5.0	0.45				6.0	0.60				8.0	0.68				10.0	1.30			
	Fresh, frozen, or processed seafood	6.0	0.30				6.5	0.43				7.0	0.68				7.5	1.10			
	Insects	3.2	0.20				3.2	0.20													
**Dairy and alternatives**	Milk (products) and plant-based milk alternatives ^1^	1.4		6.0			1.7		8.0			2.7		10.0			6.0		14.0		
	Cheese (products) and plant-based cheese alternatives	7.5	0.40				8.5	0.50				10.0	0.60				19.0	1.20	6.0		
**Oils, fats, spreads**	Oils, fats, spreads	16.0	0.10				30.0	0.35				36.0	0.52				55.0	0.75			
**Meals**	Main meals	2.0	0.24	5.0	2.4	190 and 600 ^2^	3.0	0.34	7.0	1.4	200 and 600 ^2^	4.0	0.40	10.0	1.0	225	5.0	0.53	11.0	0.8	275
	Sandwiches and rolls	2.0	0.45	5.0	2.4	190 and 350 ^2^	3.0	0.57	7.0	1.4	215 and 350 ^2^	4.0	0.62	10.0	1.0	225	5.0	0.80	11.0	0.8	275
	Soups	1.1	0.25	4.0			2.0	0.29	5.0			3.5	0.35	9.0			4.0	0.39	10.0		
**Discretionary (or non-basic) food groups**																					
**Sauces**	Meal sauces	1.1	0.40	6			1.3	0.70	8			2.5	2.20	16			6.0	4.50	26		
	Emulsified sauces	3	0.70	10		350	4.5	1.00	12		380	6	1.20	17		550	8	1.80	21		650
	Dark sauces		3.00	16				5.50	20				6.50	25.5				7.75	35		
	Other sauces (water-based)		0.75	16.0		100		0.80	25.0		130		0.90	31.0		150		1.08	39.0		190
**Snacks**	Savory snacks	4.0	0.40	4.0		500	7.0	0.79	6.5		535	9.0	0.88	9.0		540	13.0	1.00	16.0		570
	Sweet snacks	6.0	0.20	20.0		220	12.0	0.22	45.0		475	16.5	0.31	55.0		510	20.0	0.41	62.0		550
**Liquids**	Fruit and vegetable juices			5.0					8.0					10.0					11.0		
	Beverages			2.5					5.5					8.0					11.5		
**Other**	All other products	1.1 or 10 ^3^	0.10	2.5 or 10 ^3^			1.1 or 10 ^3^	0.10	2.5 or 10 ^3^												
Equivalence criteria for plant-based alternatives ^4^																					
		Protein	Ca	Fe	Zn	B2	B12														
		g	mg	mg	mg	mg	µg														
	Plant-based milk alternatives	2	100			0.1	0.3														
	Plant-based meat alternatives	19		1.5	2.6	0.1	1.3														

^1^ Plant-based alternatives need to comply with the equivalence criteria to be eligible for L1 or L2. ^2^ kcal/portion. ^3^ energy % ^4^ Plant-based alternatives are assessed against the same criteria as their animal-based counterparts. A plant-based alternative is considered nutritionally equivalent if it meets the protein threshold and all but one of the qualifying micronutrient thresholds, as tabled below. If the product does not comply with these criteria, the Choices level is restricted to L3–L5.

## Data Availability

The original contributions presented in this study are included in the article/Supplementary Materials. Further inquiries can be directed to the corresponding author.

## Supplementary Materials

The following supporting information can be downloaded at https://www.mdpi.com/article/10.3390/nu18020258/s1, Figure S1: Flowchart diagram for the study selection process to identifying current policies and recommendations from a national, regional, and global scope; Table S1: Search query used in Scopus to understand to what extent information regarding the consumption of NSSs are incorporated in health policies, recommendations, and guidelines; Table S2: Database and analysis iTFA, Excel file. Table S3: Comparison of nutrient composition in “healthier” (as defined by the Choices level 1 and 2) cheese, milk, and meat products with published literature values. Table S4: National requirements or guidelines for the fortification of plant-based milk alternatives. Table S5: National requirements or guidelines for the fortification of plant-based meat alternatives. Table S6: Illustration of how option 1 and 2 of NSS criteria would affect the scores of milk products. Table S7: Illustration of how option 1 and 2 of NSS criteria would affect the scores of beverages products.

## Abbreviations

The following abbreviations are used in this manuscript:

AI	Adequate intake
AUSNUT	Australian Food, Supplement, and Nutrient Database
BMI	Body mass index
Choices	Choices International Foundation
EFSA	European Food Safety Authority
FBDGs	Food-based dietary guidelines
FOPNL	Front-of-pack nutrition labeling
HSR	Health Star Rating
ISC	Choices International Scientific Committee
iTFAs	Industrially produced trans-fatty acids
LMICs	Low- and middle-income countries
NCDs	Non-communicable diseases
NPS(s)	Nutrient profiling system(s)
NRF	Nutrient Rich Food Index
NSSs	Non-sugar sweeteners
PAHO	Pan American Health Organization
PRI	Population Reference Intake
RCTs	Randomized controlled trials
REPLACE	WHO’s action package for eliminating industrially produced trans-fats
SAFA	Saturated fatty acids
SSBs	Sugar-sweetened beverages
TFAs	Trans-fatty acids
UNICEF	United Nations International Children’s Emergency Fund
WGI	Whole Grain Initiative
WHO	World Health Organization
