# Supplementary material for: Revision of the Choices Nutrient Profiling System
Source: Nutrients. 2026 Jan 14;18(2):258. doi: 10.3390/nu18020258 (PMC12844751; doi:10.3390/nu18020258)
Supplement: Supplementary file 1 [file nutrients-18-00258-s001.zip › nutrients-4075988-supplementary.pdf]

Table S1 Search query used in Scopus to understand to what extent information regarding the consumption of NSS are incorporated in health policies, recommendations and guidelines.

**Research question 2**	**Search query Scopus**
Search #1	TITLE-ABS-KEY ("non-nutritive sweeteners") OR TITLE-ABS-KEY ("artificial sweeteners”) OR TITLE-ABS-KEY ("no-calorie sweeteners") OR TITLE-ABS-KEY ("zero-calorie sweeteners") OR TITLE-ABS-KEY ("artificially sweetened") OR TITLE-ABS-KEY (stevia) OR TITLE-ABS-KEY (aspartame) OR TITLE-ABS-KEY (saccharin)
Search #2	TITLE (“health policy”) OR TITLE (“nutrition policy”) OR TITLE (policy) OR TITLE (guidelines) OR TITLE (recommendations) OR TITLE (restrictions) OR TITLE (taxation)
Search #3 (#1 AND #2)	(TITLE-ABS-KEY (“non-nutritive sweeteners”) OR TITLE-ABS-KEY (“artificial sweeteners”) OR TITLE-ABS-KEY (sweeteners) OR TITLE-ABS-KEY (“no-calorie sweeteners”) OR TITLE-ABS-KEY (“zero-calorie sweeteners”) OR TITLE-ABS-KEY (“artificially sweetened”) OR TITLE-ABS-KEY (stevia) OR TITLE-ABS-KEY (aspartame) OR TITLE-ABS-KEY (saccharin)) AND (TITLE (“health policy”) OR TITLE (“nutrition policy”) OR TITLE (policy) OR TITLE (guidelines) OR TITLE (recommendations) OR TITLE (restrictions) OR TITLE (taxation))

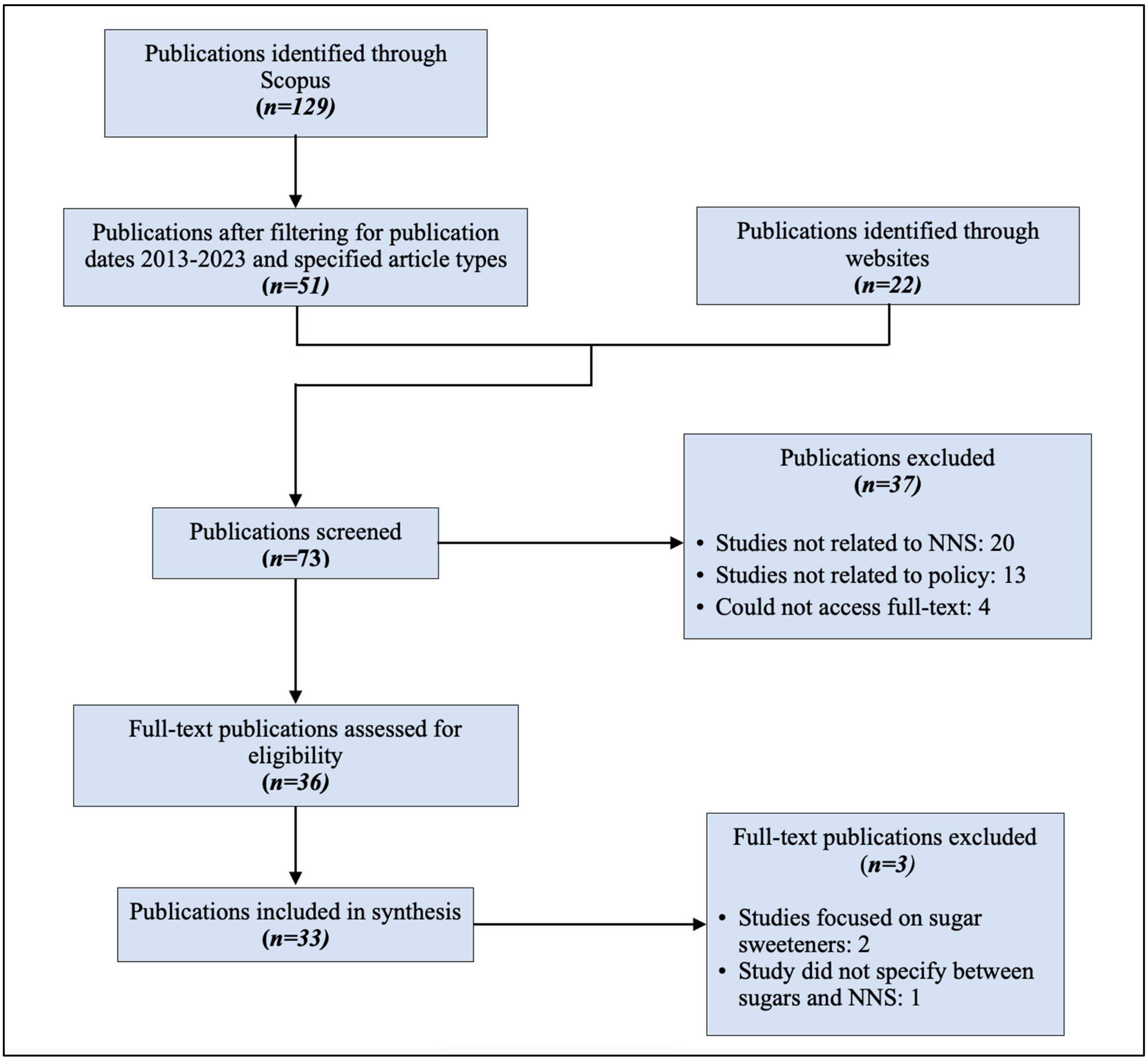

Figure S1 Flowchart diagram for the study selection process to identifying current policies and recommendations from a national, regional, and global scope.

Table S2 Database and analysis iTFA

Database and analysis iTFA	See excel file.

Table S3 Comparison of nutrient composition in “healthier” (as defined by the Choices level 1 and 2) cheese, milk, and meat products with published literature values.

Product Group	Nutrient	Database Average (“Healthier” Products)	Published Range	Notes	References
**Cheese**	Micronutrients (general)	Lower than hard cheeses (qualitative)	Varies by processing method	“Healthier” cheeses are mainly soft cheeses due to lower saturated fat and sodium. Hard cheeses generally have higher micronutrient density.	Gaucheron et al. 2011
**Milk products**	Protein (g/100 g)	4.7	2.9–3.8	Slightly higher due to high-protein yogurts.	Cimmino et al, 2023
	Vitamin A	Lower than reference milk	41 µg/100 ml	Lower because “healthier” products are lower in fat.	Cimmino et al, 2023
	Iodine (µg/l)	Within reported range	33–534	Large natural variability due to farming and processing.	Van der Reijden et al, 2017
	Other minerals and vitamins	Within reported ranges	Ca: 112–123 mg; K: 106–163 mg; etc.	Consistent with literature values for cow’s milk.	Cimmino et al, 2023
**Meat products**	Protein (g/100 g)	23	12–35	Mid-range; higher than average when all meat types included.	Pereira & Vincente, 2013
	Iron (mg/100 g)	1.9	0.5–2.4	Upper part of reported range.	Pereira & Vincente, 2013
	Zinc (mg/100 g)	3.3	0.8–3.8	Upper part of reported range.	Pereira & Vincente, 2013
	Vitamin B12 (µg/100 g)	1.4	0.4–3.0	Mid-range.	Pereira & Vincente, 2013

Table S4 National requirements or guidelines for the fortification of plant-based milk alternatives

Country / Region	Fortification Requirement	Mandatory or Voluntary?	Key Nutrients Mentioned	Notes / Sources
**United States**	No mandatory requirements; fortification encouraged	Voluntary	Ca, Vit D, Vit B12, Vit A	FDA requires clear labeling
**Canada**	Guidelines to match dairy milk nutrient profile	**Guideline (not mandatory)**	Ca, Vit D, Vit B12, Vit A, Riboflavin, Zn	Health Canada
**European Union**	Fortification allowed but not required	Voluntary	Ca, Vit D, Vit B12	Strict regulation of fortification levels
**United Kingdom**	Encourages fortification	Voluntary	Ca, Vit D, Vit B12	Clear labeling required
**Australia & New Zealand**	Calcium fortification required for beverages labelled as “milk”; vitamin B12 required if marketed as milk alternative	**Partly mandatory**	Ca (≥100 mg/100 mL), Vit B12; often Vit D	FSANZ regulations
**Brazil**	Fortification required	**Mandatory**	Ca, Vit D, Fe (sometimes)	ANVISA standards
**India**	Recommends fortification	Voluntary	Ca, Vit D, Vit B12	FSSAI
**South Africa**	Fortification required	**Mandatory**	Ca, Vit D, Vit B12	National regulation
**Saudi Arabia & UAE (GCC)**	Fortification required	**Mandatory**	Ca, Vit D, Vit B12	GCC regulatory standards
**General / Other regions**	No standard practice	Mostly voluntary	Varies by brand	U.S. and Asian markets inconsistent

Table S5 National requirements or guidelines for the fortification of plant-based meat alternatives

Country / Region	Fortification Requirement	Mandatory or Voluntary?	Key Nutrients Mentioned	Notes / Sources
**United States**	No specific requirements	Voluntary	Fe, Vit B12 (variable)	Regulated under general FDA fortification rules
**Canada**	Guidelines exist; compliance monitored	Guideline (not mandatory)	Fe, Vit B12	Regulated by Health Canada and CFIA
**European Union**	No specific mandates; must comply with fortification rules	Voluntary	Varies	EFSA regulates safety and labeling
**Australia & New Zealand**	Fortification allowed; not required	Voluntary	Fe, Vit B12 (variable)	FSANZ
**Brazil**	Guidelines for food fortification	Voluntary	Fe, Vit B12	ANVISA
**South Africa**	Interest in expanding fortification standards	Emerging / voluntary	Fe, Vit B12	Low fortification rates (<20%)
**India**	Fortification guidelines exist for multiple foods	Voluntary	Fe, Vit B12	Limited implementation for meat analogues
**Netherlands**	High voluntary fortification rates	Voluntary	Fe, Vit B12	~70% fortified with both nutrients
**United States**	High variability in fortification	Voluntary	Fe (95%), Vit B12 (24%)	Market data
**Belgium & Spain**	Moderate–high fortification rates	Voluntary	Fe, Vit B12	40–90% of products fortified
**Germany, France, Sweden**	Lower fortification rates	Voluntary	Fe, Vit B12	Consumer concerns about bioavailability
**Other regions**	No mandatory fortification	Voluntary	Varies	—

Table S6 Illustration of how option 1 and 2 of NSS criteria would affect the scores of milk products.

Choices thresholds for Milk(-products)

	**T1**	**T2**	**T3**	**T4**
SAFA g/100g	1.4	1.7	2.7	6.0
Sugar g/100g	6.0	8.0	10.0	14.0

	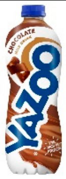	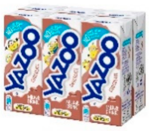	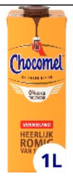
Product description	Chocolate milk drink (UHT)	Chocolate lactose reduced milk drink (UHT)	Cocoa drink based on milk (1.3% fat) and cocoa, with sweeteners (UHT)
Ingredients	Semi-Skimmed Milk, Sugar, Fat Reduced Cocoa Powder (1%), Stabilisers: Cellulose, Cellulose Gum and Carageenan, Natural Flavouring	Lactose Reduced Semi Skimmed Milk, Lactose Reduced Skimmed Milk, Fat Reduced Cocoa Powder (1.2%), Stabilisers: Cellulose, Cellulose Gum and Carrageenan: Natural Flavoring	MILK (1.3% fat), 2.1% cocoa, WHEY protein, stabilizers: cellulose, cellulose gum, carrageenan, flavor enhancer: erythritol, sweeteners: acesulfame-K, sucralose
**Nutritional Information per 100 ml**			
Energy (kJ)	275	202	224
Fat (g)	1.5	1.4	1.7
- of which saturates (g)	1.1	1.0	1.1
Carbohydrates (g)	9.1	5.0	5.3
- of which sugars (g)	8.9	4.7	5.1
Protein (g)	3.3	3.3	3.7
Salt (g)	0.1	0.1	0.15
**Choices evaluation**			
SAFA	1.1 ≤ T1 = 1.4; L1	1.0 ≤ T1 = 1.4; L1	1.1 ≤ T1 = 1.4; L1
Sugar	T2 = 8 ≤ 8.9 ≤ T3 = 10.0; L3	4.7 ≤ T1 = 6.0; L1	5.1 ≤ T1 = 6.0; L1
NNS	Not present	Not present	Present (acesulfame-K, sucralose)
**Choices Option 1**: Level / Graded 5-level FOPL	L3 / C	L1 / A	L3 / C
**Choices Option 2**: Level / Graded 5-level FOPL	L3 / C	L1 / A	L2 / B

Table S7 Illustration of how option 1 and 2 of NSS criteria would affect the scores of beverage products.

**LIQUIDS**		**T1**	**T2**	**T3**	**T4**
BEVERAGES	Sugar g/100g	2.5	5.5	8.0	11.5

	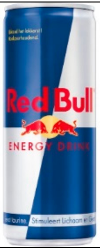	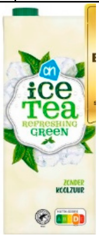	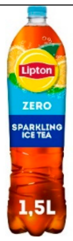
Product description	Energy drink	Non-carbonated soft drink with 0.1% green tea extract with sugar and sweetener	Refreshing drink with tea extract and sweeteners.
Ingredients	Carbonated water, sucrose, glucose, acid (citric acid), taurine (0.4%), acidity regulators (sodium carbonates, magnesium carbonates), caffeine (0.03%), vitamins (niacin, pantothenic acid, riboflavin, vitamin B6, vitamin B12) flavourings, colour (caramel).	water, sugar, acidity regulator (citric acid [E330], sodium citrates [E331]), green tea extract°, natural flavoring, antioxidant (ascorbic acid [E300]), sweetener (steviol glycosides from Stevia [E960a]).	Carbonated water, acidifiers (citric acid, malic acid), tea extract(1) (0.24%), acidity regulator (trisodium citrate), lemon juice from concentrated juice (0.1%), flavourings, antioxidant (ascorbic acid), sweeteners (sucralose, acesulfame-k).
**Nutritional Information per 100 ml**			
Energy (kJ)	195	61	20
Fat (g)	0	0	0
- of which saturates (g)	0	0	0
Carbohydrates (g)	11	3.6	0
- of which sugars (g)	11	3.6	0
Protein (g)	0	0	0
Salt (g)	0.1	0	0.29
**Choices evaluation**			
Sugar	T3 = 8 ≤ 11 ≤ T4 = 11.5; L4	T1 = 2.5 ≤ 3.6 ≤ T2 = 5.5; L2	0 ≤ T1 = 2.5; L1
NNS	Not present	Present (steviol glycosides)	Present (sucralose, acesulfame-k)

		(Not present, if Stevia would be excepted)	
**Choices Option 1**: Level / Graded 5-level FOPL	L4 / E	L3 / D<br>L2 / C (if Stevia would be excepted)	L3 / D
**Choices Option 2**: Level / Graded 5-level FOPL	L4 / E	L3 / D<br>L2 / C (if Stevia would be excepted)	L2 / C
